# Supplementary material for: Dermal Pericytes Exhibit Declined Ability to Promote Human Skin Regeneration with Ageing in 3D Organotypic Culture Models
Source: Cells. 2021 Nov 6;10(11):3051. doi: 10.3390/cells10113051 (PMC8616368; doi:10.3390/cells10113051)
Supplement: Supplementary file 1 [file cells-10-03051-s001.zip › cells-1413046-supplementary.pdf]

SAppendix B: Supplementary Figure S1:

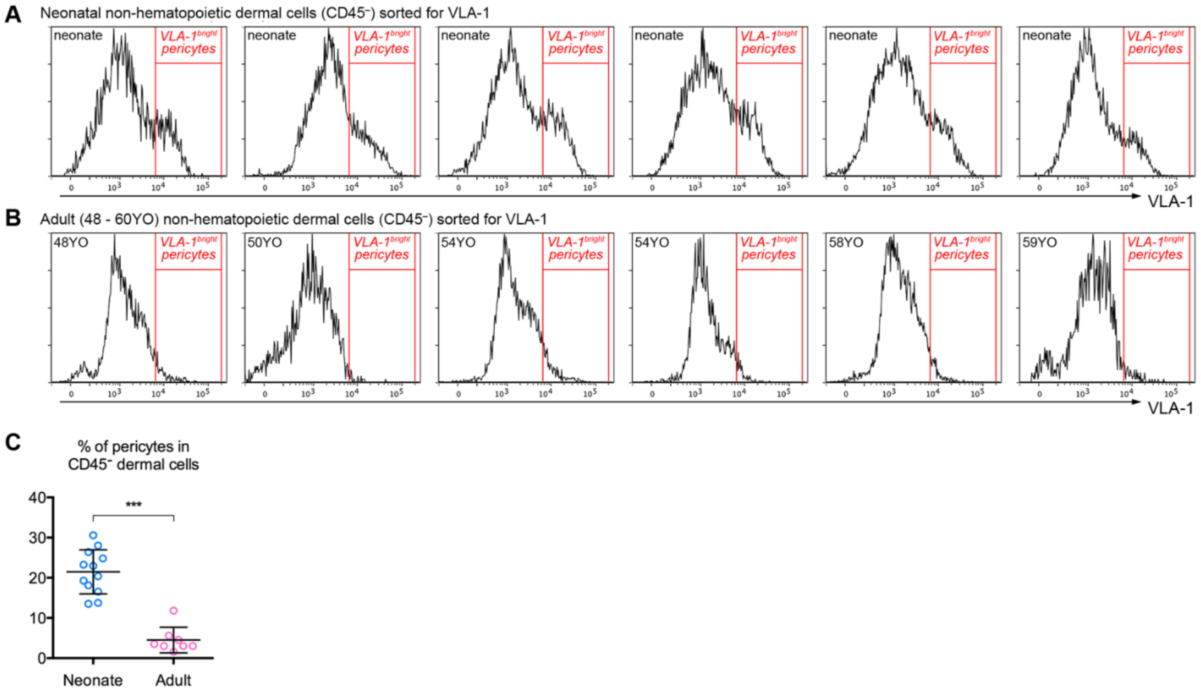

Supplementary Figure S1: Flow cytometric analysis to detect the incidence of dermal pericytes in neonatal versus aged skin. A, B. Dual staining for CD45 and CD49a (VLA-1) and analysis of the CD45^{-} fraction of dermal cells illustrates a higher incidence of CD49a^{bri} skin pericytes in neonatal foreskin (A, n=6) than aged 48-59yo adult skin (B, n=6). **C.** Quantitation of pooled data from A, B showing lower incidence of pericytes in aged skin versus neonatal skin (***=p<.001). Error bars represent mean ± SD.

Supplementary Figure S2:

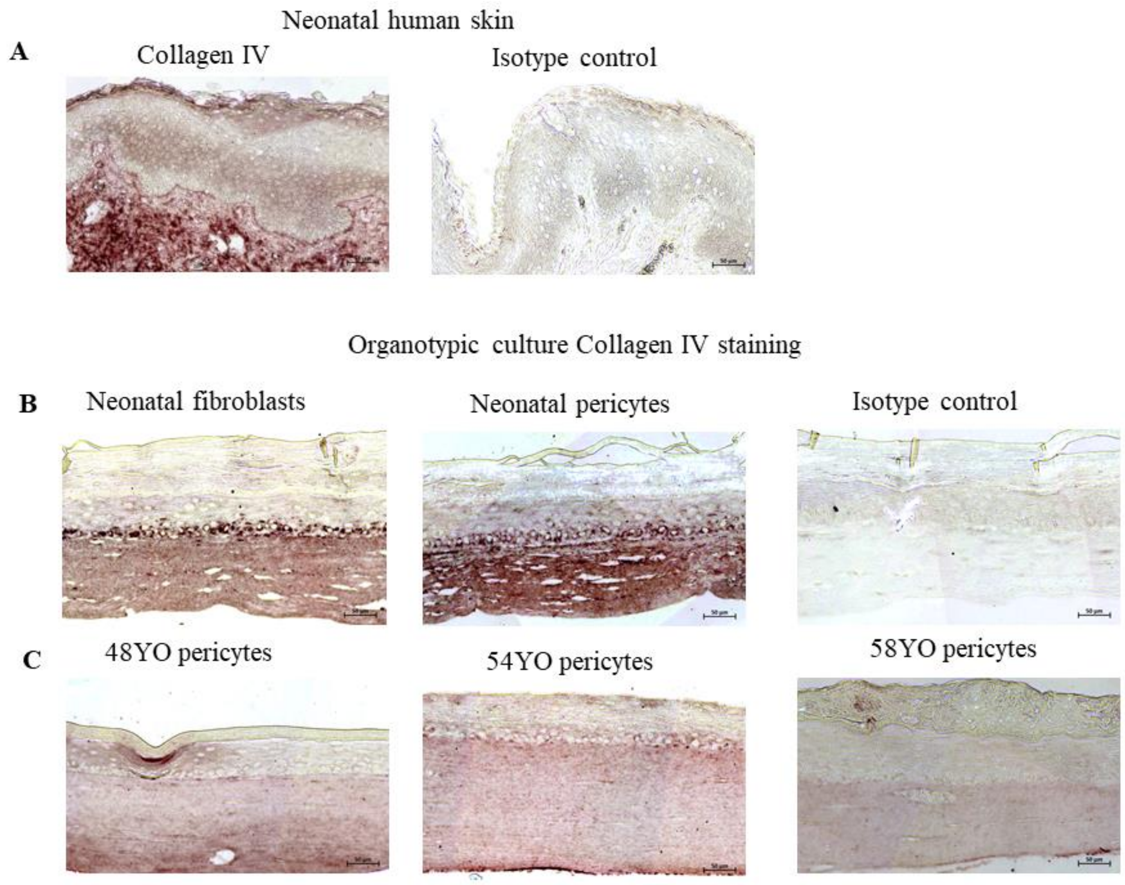

Supplementary Figure S2: Anti-Collagen IV staining of organotypic cultures populated with neonatal dermal fibroblasts, neonatal dermal pericytes or aged dermal pericytes. **A.** Anti-Collagen IV staining on neonatal human skin and rabbit IgG isotype control. **B**. Anti-Collagen IV staining on organotypic cultures with neonatal dermal pericytes or fibroblasts and rabbit IgG isotype control. **C.** Anti-Collagen IV staining organotypic cultures generated with aged pericytes from 48, 54 and 58YOI pericytes.
